# Method of Fuzzy Analysis of Qualitative-Environmental Threat in Improving Products and Processes (Fuzzy QE-FMEA)

**DOI:** 10.3390/ma16041651

**Published:** 2023-02-16

**Authors:** Andrzej Pacana, Dominika Siwiec

**Affiliations:** Faculty of Mechanical Engineering and Aeronautics, Rzeszow University of Technology, al. Powstancow Warszawy 12, 35-959 Rzeszow, Poland

**Keywords:** quality, production processes, customers requirements, FMEA, decision support, fuzzy decision making, mechanical engineering, sustainability, impact on natural environment, production engineering

## Abstract

Improving products and production processes is necessary to ensure the competitiveness of the organisation. As part of these improvements, the popular approach is to use the FMEA method (Failure Mode and Effect Analysis). In the traditional FMEA approach, only the qualitative aspect is included, i.e., the analysis of the quality level of products or processes, its possible incompatibilities, and then proposing improving actions for them. It seems insufficient in times of expansion of the idea of sustainable development and dynamically changing customer requirements. Hence, the purpose of the research is to develop a fuzzy QE-FMEA method to simultaneously analyze hazards for product quality and the natural environment. This method will be based on a fuzzy decision environment. The main elements of originality of the developed method are: (i) extension of the characteristics of the selection of ratings for indicators with triangular fuzzy numbers and the development of a new characteristics of the selection of ratings for the environmental impact indicator, (ii) development of a selection matrix for the qualitative-environmental indicator (QE) according to the rules of triangular fuzzy numbers, (iii) determination of the method of estimating the value of the threat priority, additionally considering the qualitative-environmental indicator (RQE). The complement of research is developed procedure of the Fuzzy QE-FMEA method. It was shown that it is possible to include the effects of incompatibilities (effects of defects occurring in products or processes), which were simultaneously assessed considering the importance and impact on the natural environment. This method will be useful for any company for analysing defects of any products or processes mainly with significant impact on the natural environment.

## 1. Introduction

The products and processes of their occurrence require continuous improvement. It is a necessary action for all prosperous enterprises [1,2]. In order to stabilise actions made by enterprises, it is important to effectively analyse hazards in the area of projects, processes, and products [3,4]. These thoughtful actions could reduce the incompatibilities of products or processes; hence, its quality is more sufficient. This approach is in line with the idea of continuous improvement. Furthermore, to pursue sustainable development and production, it is necessary to include the impact of these actions on the natural environment and [5,6,7]. It results from the fact that currently popular and necessary are taking actions to improve products and processes in this way to be in accordance with the principles of sustainable production, which in the case of production enterprises refers to, for example, the analysis of new technology, the achievement of savings in production and the reduction in negative impact actions of production on the natural environment [8,9,10]. Therefore, enterprises strive to reduce the waste of production resources, and try to take into account customer expectations on an ongoing basis and to care for the natural environment in their activities [11,12]. However, continuously taking improving actions should be supported by adequate techniques because even in stable products or processes, which could occur new, hitherto unknown, defects, failures, and incompatibilities. Therefore, companies should use techniques that will support their analysis and, consequently, elimination. One of the most popular uses for analysing products or industrial processes is the FMEA method (Failure Mode and Effect Analysis) [13,14,15], so the technique of systematic process to identify potential failures and the causes and effects of their occurrence to limit and eliminate them. The FMEA method comes from the automotive industry, but it is applicable at various stages of the life of any product or process. Its use supports the improvement of the products and processes of its occurrence, among others, by improving the design process and production [16,17]. It was observed that the FMEA method was not used for the analysis of hazards in products and processes, considering simultaneously the effects of incompatibility of the quality of products or processes and their impact on the natural environment. In this approach, the quality of products (or processes) refers to their ability to meet customers’ expectations. Quality is a degree of product compliance with recipients’ requirements. Therefore, it is necessary to analyse different aspects of the quality of products at different stages of processes of their creation. In turn, the impact on the natural environment is each action of the company that has a direct or indirect impact on the natural environment. To reduce threats, it is necessary to analyse mainly the negative influences on products or processes. The improving the actions of products or processes are usually aimed to meeting customer satisfaction, which is identified with the high quality of products or processes. However, the negative impacts on the natural environment of these improving actions are often omitted because of high quality. According to the idea of the sustainable development, it is necessary to combine these two factors, i.e.: quality and environmental impact. However, the popular in practise the popular FMEA method is not adjusted for that. It was considered a research gap, which was filled by the proposed Fuzzy QE-FMEA method.

For example, in the study [18] the authors analysed failures that impact the quality of castings in the foundry industry, for example motor housing. In this aim, the combined methods were used, i.e., the fuzzy analytical hierarchical process and fuzzy TOPSIS-based FMEA analysis. These methods worked in a fuzzy decision environment. In this work, the quality aspect was analysed, but the impact on the environment was omitted. However, authors of work [19], analysed the possibility of including cleaner production as part of the use of the FMEA method. This was mainly carried out to prevent machine failure. In turn, in this work, the environmental aspect was analysed but was not linked to quality aspects. Similarly, in, the authors of the article [20] used the FMEA method in the area of reducing the negative impact on the natural environment, but the quality aspects of the processes were omitted. In this case, the FMEA method was used to analyse processes aimed at the formation of hazardous waste, sewage. the aspects of existing industrial laboratories were assessed and their impact on the natural environment was assessed. In turn, in terms of only quality (omitted impact on the natural environment), the FMEA method was used to evaluate, e.g., products from the automotive industry [21] or medical [22]. Further examples are studies, that is, [23,24,25,26]. Among others, the study [23] analysed risk in the development of a new product (machined part). The methods used were FMEA and Pythagorean fuzzy-dimensional analysis (PFDA). The authors of the study [24] analysed quality problems in production in the production hale, analysing the effects of failures for different processes and products, within problems with resources and a way of design. In the context of quality of processes, an analysis was carried out according to the proposed FMEA-linked-to-PPR model, so the model analyses the problem with resources (FPI). In this case, the impact on the natural environment was omitted. Another example in the same context shown in the study [25], in which the proposed approach was based on the FMEA method, where cause-and-effect relationships were included to assess the risk of unit production. The tools that were used were as follows. sequential multistage fuzzy cognitive maps (MSFCMs) and learning algorithm. The subject of the research was the production process of automobile parts. Whereas, to analyse the quality of products and processes, the authors of the study [26], analysed selected accidents in the automotive industry to allow minimalization of quality errors and increased efficiency in production. The 8D method, which is described in Ref., e.g., [26], was used for this, but an analysis was carried out using value stream mapping (VSM) or impact and failure analysis (FMEA). In this case, the impact on the natural environment was also omitted, and the analysis was performed only for quality aspects.

After a review of the literature, it was shown that the FMEA method is applicable to the analysis of defects in various products or processes. It was modified or combined with other methods. However, no such approach was found where the FMEA method would take into account the effects of incompatibility of quality products or processes and simultaneously their impact on the natural environment. This was considered a research gap that was assumed to be filled.

The purpose of the research is to develop a method of fuzzy analysis of qualitative environmental hazards to improve products and processes (Fuzzy QE-FEMA), which will function in a fuzzy decision environment. The method was created by modifying the FMEA method so that it ensures simultaneous consideration of the effects of quality discrepancies (in products or their creation processes) and their impact on the natural environment, where the proposed method will function in a fuzzy decision-making environment. In the proposed approach, the quality is the importance of the defect for the customer. For this purpose, the following hypothesis was adopted:

**Hypothesis 1.**  *In improving products and processes using the FMEA method, it is possible to simultaneously include the effects of incompatibilities in view of quality and impact on the natural environment, which will be supported by a fuzzy decision environment.*

Originality is to develop a new approach to use the FMEA method by including the effects of incompatibilities of both quality and impact on the natural environment. The Risk Priority Number (RPN) will be derived from the qualitative and financial effects and the impact of these effects on the natural environment. Other priority numbers will be selected as described in the subject of literature, especially, i.e., [27]. Moreover, a novelty of the study is proposed to include in the FMEA, the qualitative-environmental index according to the theory of fuzzy decision environmental (triangular fuzzy numbers), which as shown by the authors of studies, e.g., [28,29] allows for the achievement of a higher precision of decision-making. The motivation of the implemented fuzzy numbers resulted from a new approach to include in the FMEA method the impact on the natural environment. It causes decisions made so far in terms of the quality of products and processes may be less precise (uncertain). The fuzzy logic approach allows for reducing this uncertainty and simultaneously allows for more effectiveness of the proposed fuzzy QE-FMEA method. Furthermore, it could be useful in making right improving actions.

The research for developing the Fuzzy QE-FMEA method was shown in the following way: the motivation, concept, and assumptions of the Fuzzy QE-FMEA method, description of the developed method, the procedure of the method, and results.

## 2. Justification and Conditions for the Originality of the Method

### 2.1. Motivation and Concept of Fuzzy QE-FMEA Method

**Figure 1:**
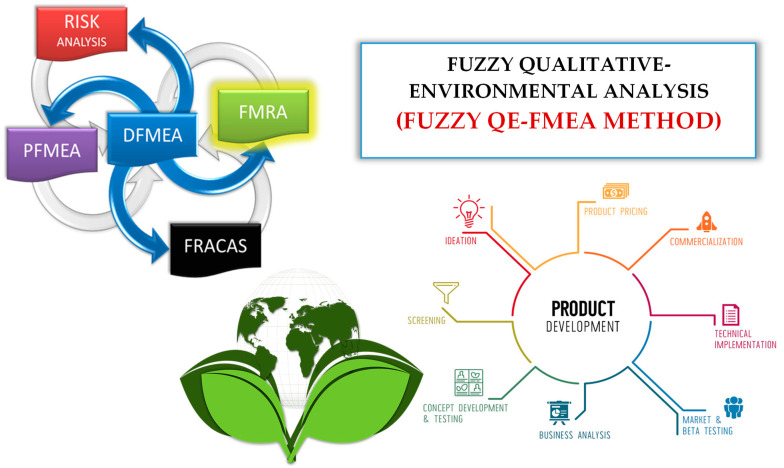
Workflow in the Fuzzy QE-FMEA method.

As part of the research, a method was developed, written with the acronym Fuzzy QE-FMEA, which is a modification of the FMEA method (Failure Mode and Effect Ana-lysis), otherwise known as the FMECA method (Failure Mode and Cricitality Analisys) or the AMDEC method (Analys des Modes de Defaillace et Leurs Effects) [2,30]. Motivation for the research resulted from a review of the literature on the subject and specific research gaps. Therefore, the process of developing the fuzzy QE-FMEA method was the process of modifying (improving) the FMEA method to achieve a simultaneous analysis of the consequences of incompatibility in terms of product (or process) quality and their impact on the environment. The method is to contribute to the increase in customer satisfaction and to the reduction in the negative impact on the natural environment. This method was called “Fuzzy QE-FMEA”, because it was developed on the basis of the traditional approach of the FMEA method. This method was created based on rules of fuzzy logic, and hence the “fuzzy” prefix was added to the name of this method. In turn “QE” means, in short, “Q” as qualitative threats and “E” as environmental threats, which were combined in this method. Its general concept is presented in Figure 1.

In the traditional FMEA approach, there is a risk priority which is the product of indicators, i.e., the probability of a defect (P), the significance of the defect (Z) and the possibility of detecting the defect (W). However, the FMEA methodology does not ensure the simultaneous consideration of the effects of incompatibility of quality products or processes and their impact on the natural environment.

For this reason, the motivation for the research was to modify the FMEA method to make it possible. Therefore, it was assumed to include additional indicators of impact on the natural environment (E), for which a new scale of assessment was described in triangular fuzzy numbers and simultaneously in the traditional scale from 1 to 10. The indicator of impact on the natural environment is integrated with the indicator of the effect (importance) of the defect (Z) on the quality of the product or process and, according to it, an it so-called qualitative-environmental indicator (QE) is created. This index was marked “Q” index because in this approach it is combined with the “E” index (environmental impact). In the traditional approach, the “Z” index is not combined with the “E” index”. Therefore, to distinguish these differences, these indexes were marked in this way. Integration is carried out in a pairwise comparison matrix specially developed for this purpose. The matrix was created according to the principle of triangular fuzzy numbers. This is due to the fact that the indicators in the FMEA method are subjectively measured on a scale of 1 to 10. However, as mentioned by the authors of the study [31], these indicators are ordinal scale variables that have a rank, but the distances between them are not measured. This is due to the lack of implementation of the function that determines the distance between them in the FMEA method. For this reason, it was assumed that the improvement of the indicator selection FMEA method with the method in this method will be implemented by introducing triangular fuzzy numbers (Saaty scale). According to the authors of the works [28,29,32,33], these numbers are more effective than the numbers on an ordinal scale, because they ensure greater precision of decisions by reducing uncertainty and subjectivity in expert assessments. Therefore, fuzzy numbers were used to develop a selection matrix for the qualitative environmental indicator (QE), whose assessments function in a fuzzy decision-making environment [12,34,35,36].The developed matrix is dedicated to the proposed Fuzzy QE-FMEA method. On its basis, the QE value is selected, which replaces the traditional Z indicator. In the proposed approach, the value of the risk priority is marked as (RQE), where it is the product of the value of the defect probability index (P), the defect detection index (W) and the qualitative-environmental index (QE). Based on the RQE, the risk is determined, taking into account the effects of quality incompatibility, and the impact on the natural environment.

### 2.2. Assumptions of Fuzzy QE-FMEA Method

The assumptions were assumed based on the FMEA methodology, assumptions for decision making based on multiple criteria in a fuzzy decision environment, and also based on the literature review. The assumptions for the modified FMEA method, i.e., for fuzzy QE-FMEA method were following:

- products or processes to be analysed are arbitrary with the use of adequate priority number selection tables [1,3,37];
- quality is expressed by the Z index (i.e., the importance of the defect for the customer) and refers to the effect of the defect on the use of the product/or the functioning of the process, the impact on customer satisfaction and possible repair costs) [7,29,38];
- the impact on the natural environment (indicator E) is the negative impact of a defect (product or process) on the natural environment [4,12,38];
- the threat priority value (RQE) is calculated in a fuzzy decision-making environment and is a quotient of the ratings assigned to the indicators, i.e.,: P—probability of a defect, W—possibility of detecting a defect, Z—effect (significance) of the defect (so-called quality), E—impact on the natural environment, where Z and E are combined and created qualitative environmental indicator (QE) and are evaluated simultaneously in the pairwise comparison matrix [2,30,39,40];
- when determining the number Z (significance of the defect), only the effect of the defect should be considered;
- determining the number P (the probability of a defect) may refer to the defect but also to the cause of the defect, it is necessary to consistently comply with the adopted rule;
- determining the number W (the possibility of detecting a defect) refers to the cause of the defect;
- criteria for assessing indicators P (probability of a defect), W (possibility of detecting defects), and QE (qualitative-environmental indicator) result from the subject of the analysis and are selected individually by a team of experts, considering, for example, the frequency of incompatibilities, their effects, and causes;
- the QE indicator results from the relationships occurring in a fuzzy decision-making environment and the nine-point Saaty scale [3,12,33].

The assumptions adopted were the basis for improving the FMEA method, which was called the fuzzy QE-FMEA method.

## 3. Description of Fuzzy QE-FMEA Method

The way of modification of the FMEA method to develop the Fuzzy QE-FMEA method included:

stage 1: extension of the characteristics of the selection of ratings for the P, W, Q indicators with triangular fuzzy numbers and the development of new characteristics of the selection of ratings for the environmental impact indicator,
stage 2: development of a selection matrix for the qualitative-environmental indicator (QE) according to the rules of triangular fuzzy numbers,
stage 3: determining the method to estimate the value of the threat priority, also considering the qualitative-environmental indicator (RQE).

The indicated main stages of improving the FMEA method are characterized in the following part of the study.

### 3.1. Extending the Characteristics of the Selection of Assessments for Indicators in the Fuzzy QE-FMEA Method

**Figure 2:**
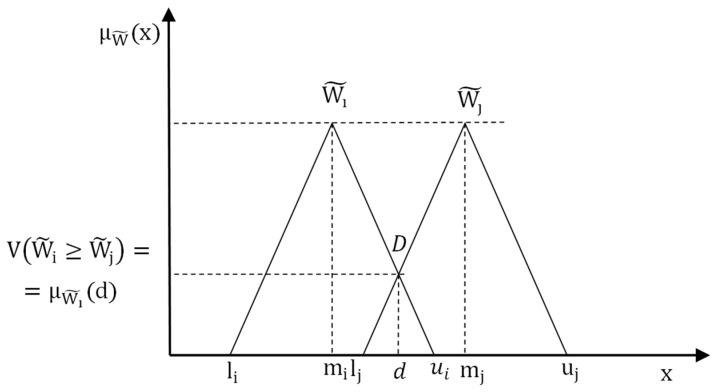
Determination of the coordinates of the point of intersection ${\tilde{W}}_{i}$ and ${\tilde{W}}_{j}$.

Traditional characteristics of a selection of indicators used in the proposed method, that is, characterisation of indicators P, W, and Q. Each characteristic is extended by assessment in triangular fuzzy numbers to show the dependence between traditional and triangular assessment of indicators in the FMEA method. The characteristics of triangular fuzzy numbers $\left(\tilde{W}\right)$ are shown, e.g., in [33,36,41]. These numbers are described by three elements $\left(l_{\mathrm{ij}},m_{\mathrm{ij}},u_{\mathrm{ij}}\right)$. Figure 2 shows two fuzzy numbers $\tilde{W_{i}}=\left(l_{\mathrm{ij}},m_{\mathrm{ij}},u_{\mathrm{ij}}\right)$ and $\tilde{W_{j}}=\left(l_{\mathrm{ji}},m_{\mathrm{ji}},u_{\mathrm{ji}}\right)$, where $\mu_{\tilde{W_{i}}}\left(d\right)$ is degree of belonging to $\tilde{W_{i}}$ (Figure 2).

The proposed dependence between the traditional scale (ordinal numbers) and Saaty’s scale (fuzzy number) is shown in the next stage, i.e., during the development matrix to select the assessment of qualitative-environmental indicator (QE). Therefore, at this stage, it is necessary to develop the characteristics of the selected assessments for a new indicator E, i.e., impact on the natural environment. Developed initial tables of selected indicators for process in the fuzzy QE-FMEA method, as shown in Table 1, Table 2, Table 3 and Table 4. In turn, tables are presented in the literature to select these indicators for a product, e.g., [27,31,42,43,44].

The fuzzy numbers for indicators for the product should be used according to characteristic tables for the product. The method is similar to tables for the process.

### 3.2. Development of a Matrix to Select Qualitative-Environmental Indicator (QE)

In the developed method Fuzzy QE-FMEA, it was assumed that the evaluation effects of incompatibilities will be realised based on a qualitative-environmental indicator, which simultaneously determines quality Q (advantage defect for the customer) and the impact on the natural environment (E). Therefore, the next stage of the methodology was to develop this matrix, as shown in four main steps.

#### 3.2.1. Calculation of Summary Values of Quality and Environmental Impact Assessments

First, the sum of the assessment values that refer to quality (Q) and impact on the natural environment (E) is estimated. These values are determined on a scale of 1 to 10 (Table 3 and Table 4). Following the assumption of the authors of the work [3,12,33] assumed, that sum of values $a_{\mathrm{ij}}$) are noted in the pairwise comparison matrix $M_{\mathrm{ij}}^{1}={\left[a_{\mathrm{ij}}\right]}_{1\times 10}$ as shown formula (1):

(1) $$a_{\mathrm{ij}}={\;Q}_{i}+E_{j}$$

where: Q—quality, E—impact on the natural environment, i, j = 1, 2, …, 10.

The matrix $M_{ij}^{1}$ obtained after using Formula (1) has the sum of quality and impact on the natural environment, as shown in Table 5.

#### 3.2.2. Calculation of the Quotient Value of the Sum of the Assessments of Quality Indicators and Environmental Impact

Next, the quotient of the sum of the assessments ($a_{\mathrm{ij}}$) is calculated, and two summed scores Q and E, i.e.: quality assessments (Q) and impact on the natural environment (E). It is denoted by the value ($b_{\mathrm{ij}})$ and noted in matrix $M_{\mathrm{ij}}^{2}={\left[b_{\mathrm{ij}}\right]}_{1\times 10}$ as shown in formula (2):

(2) $$b_{\mathrm{ij}}=\frac{a_{\mathrm{ij}}}{2}$$

where: a—sum of assessments of quality (Q), and impact on the natural environment (E), i, j = 1, 2, …, 10.

Obtained on the rating scale from 1 to 10 values from matrix $M_{ij}^{2}$, which were calculated according to Formula (2) are shown in Table 6.

The rating scale in the FMEA method ranges from 1 to 10. Therefore, it was necessary to reduce the obtained values from $M_{\mathrm{ij}}^{2}$ matrix to integers. For this purpose, a fuzzy decision environment was used, as shown in the next step of the method.

#### 3.2.3. Determine Assessments of Qualitative-Environmental Indicators Based on Fuzzy Decision Environment

The values from the $M_{\mathrm{ij}}^{2}$ the matrix were not integers, which in the FMEA methodology are numbered 1 to 10. Therefore, the authors of the studies [28,29,32], assumed that the numbers from matrix $M_{\mathrm{ij}}^{2}$ as values of triangular fuzzy numbers. In view of the range of matrix values $M_{\mathrm{ij}}^{2}$ <1, 10> used a fuzzy nine-point classification scale. For the purposes of the developed method, this scale was extended about an additional range of triangular fuzzy numbers for number 10 [33,35,36]. Triangular fuzzy numbers with corresponding values from 1 to 10 are shown in the tables for selected indicators (i.e., Table 1, Table 2, Table 3 and Table 4). Therefore, according to the fuzzy decision environment, the values from the $M_{\mathrm{ij}}^{2}$ the matrix were expressed in triangular fuzzy numbers. The results are shown in Table 7.

Based on fuzzy matrix $M_{ij}^{\mathrm{Fuzzy}}$, the matrix for selecting qualitative-environmental indicators (QE) on an ordinal scale was developed.

#### 3.2.4. Selection Matrix of the Qualitative-Environmental Indicator (QE)

The matrix of selection qualitative-environmental indicator (QE) was called $M_{\mathrm{ij}}^{\mathrm{QE}}$ matrix. In this matrix, values of assessments were expressed on an ordinal scale, which corresponded to the values in the fuzzy matrix $M_{\mathrm{ij}}^{\mathrm{Fuzzy}}$. The purpose was to unify the scale for all indicators (P, W, Q, and E) to an ordinal scale from 1 to 10, to determine a risk priority value (RQE) according to the traditional methodology of the FMEA method [2,13,16,45]. The developed matrix of selected qualitative-environmental indicators for the Fuzzy QE-FMEA method is shown in Table 8.

Based on $M_{\mathrm{ij}}^{\mathrm{QE}}$ matrix, the choice of assessment refers to simultaneous quality and impact on the natural environment. The assessments from $M_{\mathrm{ij}}^{\mathrm{QE}}$ matrix, which are selected in the Fuzzy QE-FMEA method are marked as QE indicators. Based on the developed indicators and assessment selection matrix, it is possible to calculate the value of the threat priority, as presented in the next step of the method.

### 3.3. Determine Threat Priority Value (RQE)

In the proposed approach, the threat priority value (RQE) is calculated as the quotient of the assessments assigned to the indicators in the fuzzy QE-FMEA method, as shown in Formula (3):

(3) RQE=P×W×QE

Indicator assessments are adequate: P—probability of defect, W—possibility of defect detection, Q—the effect (significance) of the defect (so-called quality), E—impact on the natural environment, where Q and E are combined and create qualitative-environmental indicators (QE) [30,39,40].

## 4. Fuzzy QE-FMEA Procedure

**Figure 3:**
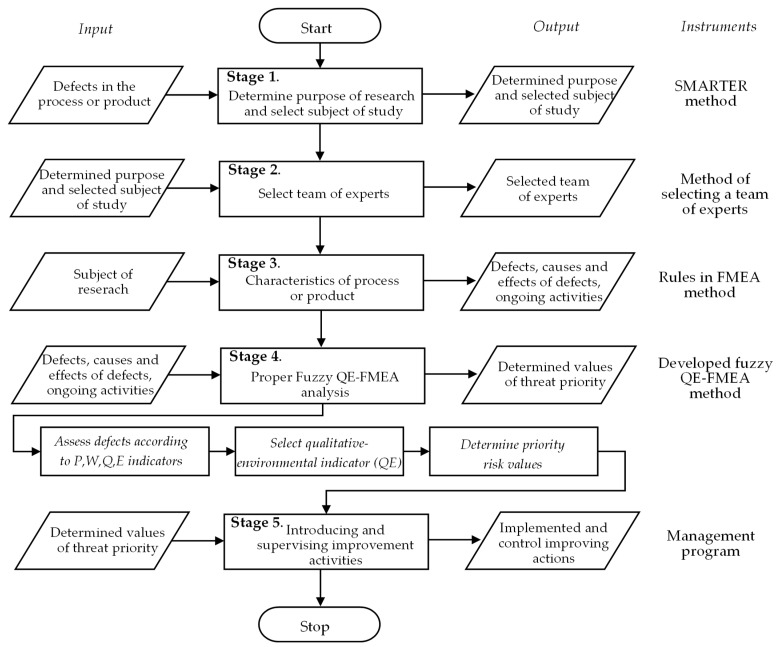
Algorithm of procedure of Fuzzy QE-FMEA method.

The procedure for the proposed Fuzzy QE-FMEA method was developed in five main stages, as shown in the algorithm of the method (Figure 3).

The characteristic of the procedure is shown in the next part of the study.

### 4.1. Determine Purpose of Research and Select Subject of Study

Firstly, the purpose should be determined. The purpose is determined by the entity (expert), for example, according to the SMARTER method [46]. In the proposed approach, the purpose should refer to improving the products and processes of its occurrence by successively eliminating the causes of defects. The general idea refers to an increase in customer satisfaction with products or products due to their occurrence and the limitation of the negative impact on the natural environment. The overarching goal is also to avoid the occurrence of recognised and unrecognised defects in new processes and structures according to the analyses carried out.

Hence, as part of the subject of research, it is necessary to make choices about products or processes. The choice is made by the entity (expert). In view that the defects of a product can refer to function, reliability, or construction technology, the choice of a product can result from, e.g., the introduction of a new product, or most modified parts of the product, or using new technology or initiation of new possibilities using the product. In turn, the FMEA for process refers to problems that occur during the realisation of construction requirements or significant impacts on the production process, for example, the processing method, processing parameters, or measurement and control measures. Therefore, the choice of process can refer to processes in the initial design phase and also before starting series production or stabilising series production. A tool that supports the choice of product or process is brainstorming (BM) [47] among the expert team, and using Pareto-Lorenz analysis, as shown in studies [48,49].

### 4.2. Select Team of Experts

The realisation of the FMEA method requires the appointment of a team of experts, which will be responsible for achieving the purpose of research. Therefore, at this stage, a team of experts was appointed to approach a given analysis individually. For this reason, it is important that the team of experts includes competent members who know the problem to be solved. The method of selecting a team of experts was presented in Ref. [37].

### 4.3. Characteristics of Process or Product

According to the assumed subject of the investigation (from Section 4.1) it is necessary to prepare the data for analysis. It relies on characterising the process or product considering stages/function of the process or elements/function of the product. Later, for each stage/function of the process (or elements/function of the product), the defects must be determined, as well as causes and effects of them [2,31,43]. In addition, it is recommended to determine the responsibility for ongoing controls, e.g., indication of the department of the enterprise. This is carried out by a team of experts as part of brainstorming.

### 4.4. Proper Fuzzy QE-FMEA analysis

At this stage, a proper analysis according to the developed Fuzzy QE-FMEA method is realised (as shown in the third chapter of the article). First, for defects determined in their process or product, it is necessary to select indicators assessment according to the proposed method, i.e., P, W, Q, E. All assessments can be determined by ordinal numbers. The evaluations are selected by a team of experts according to the characteristic tables developed for of these indicators, as shown in the third chapter (stage 1). Furthermore, according to the assumptions of the developed method, the assessment of the effects of incompatibility is realised based on a qualitative-environmental indicator, which determines simultaneously the quality Q (importance of the defect for the customer) and the impacts on the natural environment (E). The value of the qualitative-environmental indicator (QE) is selected by a team of experts according to the developed matrix $M_{ij}^{QE}$, which is shown in the third chapter of the article (stage 2). After selecting all indicators’ assessments, it is possible to calculate priority threat values (RQE). This value is calculated according to Formula (3), as shown in the third chapter of the article (stage 3).

### 4.5. Introducing and Supervising Improvement Activities

The priority threat values (RQE) should be in the range of 1 to 1000. In another case, it is necessary to repeat the analysis in the Fuzzy QE-FMEA, until the correct results are obtained. On the basis of RQE values, the ranking of causes (or defects) is created in view of their criticality. The higher the RQE score, the greater the importance of the cause or defect (Table 9).

The acceptable risk level is assumed to be 125 [44,50]. If this value is exceeded, it is necessary to take improved actions to eliminate or reduce error. Improving actions are selected by a team of experts, e.g., as part of brainstorming (BM). After the implementation of improvement actions, the Fuzzy QE-FMEA analysis should be performed again, which should show a decrease in the RQE value for critical causes or defects.

## 5. Results

The Fuzzy QE-FMEA method was performed for the process of a five-layer stretch film for manual packaging. The stretch film is produced by the largest producer of stretch film in Poland and one of the largest in Europe. The choice of the process of production of stretch foil resulted from the popularity of this product in general use and the negative impact of the production process of the stretch foil on the natural environment. Stretch film is a product that is most commonly used in the world to pack different types of products. Its main advantages are protection against damage to products and also against moisture or water and possible contamination. Stretch film is elastic, tear resistant, and also adheres well, so it has a wide range of applications [14,15]. In view of the global scale of its production, it is possible to observe its potential negative impact on the natural environment, e.g., in view of the difficulties in recovering and recycling. Therefore, as part of sustainable development, the works seem purposeful, which will be referred to improve the stretch film, and simultaneously tools supporting sustainable development production of stretch film for sustainable development to reduce the negative impact on the natural environment [15,42].

### 5.1. Determine Purpose of Research and Select Subject of Study

The process of production of five-layer stretch film for manual packaging was selected as the subject of the research. The subject of research was selected by the expert (entity) in view of the defects in this process and also of the negative impact of this process on the natural environment. The purpose of the investigation determined the entity (expert) according to the SMARTER method. In this case, the purpose of production was to improve the process of five-layer stretch film for manual packaging to successfully eliminate the causes of defects in this process. The purpose is to increase the satisfaction of stretch film customers and reduce the negative impact on the environment. The overarching goal is to avoid the occurrence of recognised and unrecognised defects in this process.

### 5.2. Select Team of Experts

According to the concept of research, a team of experts was selected. This team analysed the process of production of five-layer stretch film for manual packaging. The choice of the expert team was made according to the method shown in the study [37].

### 5.3. Characteristics of Process or Product

According to the selected subject of the study (i.e., the process of production of five-layer stretch film for manual packaging), it is necessary to prepare data for analysis. It consisted of a team of experts considering the function of the process. Later, for each function of the stretch film production process, defects were determined, and also their causes and effects were determined. The result is presented in Table 10.

Based on the process data, the next stage of the procedure was carried out.

### 5.4. Proper Fuzzy QE-FMEA Analysis

At this stage, proper analysis was performed according to the fuzzy QE-FMEA method. The team of experts selected assessments of P, W, Q, and E indicators for defects determined in the stretch film production process. All assessments were selected according to the developed characteristics tables for these indicators.

As assumed, assessments of the effects of incompatibilities were realised considering qualitative-environmental indicators, which determined simultaneously quality Q (importance defect for the customer) and impact on the natural environment (E). The value of the qualitative-environmental indicator (QE) was selected by the team of experts according to the matrix dedicated for this method $M_{ij}^{QE}$ matrix (and the corresponding matrix $M_{ij}^{\mathrm{Fuzzy}}$ with fuzzy numbers-Table 8). Next, using Formula (3), the threat priority (RQE) values were calculated. The results are shown in Table 11.

Calculations performed using the fuzzy QE-FMEA method allowed determining the threat priority (RQE), which simultaneously included the impact on the natural environment. The values of RQE were in the range from 1 to 1000, therefore, calculations were found to be correct.

### 5.5. Introducing and Supervising Improvement Activities

Based on the RQE value, a ranking of defects in the stretch film production process was created. Furthermore, the acceptable risk level was assumed to be 125. For defects whose RQE value exceeded this value, improvement actions were proposed. The actions were defined by a team of experts after brainstorming (BM). They are presented in Table 12.

Several incompatibilities were with a value RQE < 125. Therefore, due to these incompatibilities, there is no need to take improvement actions. For other incompatibilities, these actions were proposed. After implementing these improved actions, it is necessary to perform another analysis with the fuzzy QE-FMEA method. In the future, this analysis should show a reduction in the RQE value for critical causes of incompatibilities. It is the last stage of the proposed method.

## 6. Discussion

Improving the quality of products and their occurrence processes is a challenge [51,52], especially in the era of dynamic changes in customer expectations and an increased impact on the natural environment [53,54,55,56]. There is a search for different solutions that support an effective quality management. One of them is, for example, the popular FMEA method, which can be used as part of causes and effects analysis [57,58]. However, this method in the traditional approach does not take into account the impact on the natural environment. In view of this, the fuzzy method of qualitative-environmental risk analysis was developed to improve products and processes (Fuzzy QE-FMEA).

This method was created as the modified FMEA method, to allow the simultaneous inclusion of the effects of qualitative incompatibilities and their impact on the natural environment. A test of the method was carried out for the production process of a five-layer stretch film for manual packaging. The results obtained by the traditional approach of the FMEA method to the proposed approach of the Fuzzy QE-FMEA method are shown in Table 13.

The method test and comparison of the traditional approach with the new one allowed us to confirm the hypothesis that, when improving products and processes using the FMEA method, it is possible to include simultaneously the effects of incompatibilities in view of quality and impact on the natural environment, which will be supported by a fuzzy decision environment After the comparison, it was observed that for some defects, replacing the Q indicator with the QE indicator did not affect the value of the hazard indicator, for example, improper setting of the technological line (RPN = RQE = 180) or low-quality raw material (RPN = RQE = 120). It showed that the assessments of the Q and QE indicators were the same. It mainly resulted in little importance of the impact on the natural environment of the causes of these incompatibilities. Although, when the causes of incompatibilities had a significant impact on the natural environment, a change in the RPN indicator was observed compared to the RQE indicator. It was an increase in the RPN index, where the greater the impact on the environment, the greater the increase in the indicator. In view of that, the applied proposed method (Fuzzy QE-FMEA) could increase the number of necessary actions to be launched in the event of the need to take improvement actions, i.e., when the RQE indicator was greater than 125. In this approach, it was concluded that the proposed method can be useful due to the need to take more improvement actions, which resulted from the possibilities to achieve higher RQE ratios, so the need simultaneously includes not only the probability of defect occurrence, the possibilities its detection, or importance of defect (i.e., quality), but also impact on the natural environment.

## 7. Conclusions

The products and processes of their production need continuous improvement to effectively analyse risk in the area of projects, processes, and products considering their impact on the natural environment. This is due to the fact that it is popular and necessary to take action to improve products and processes in such a way that they are consistent with the principles of sustainable production.

Hence, the purpose of the research was to propose an improved FMEA method, which will operate in a fuzzy decision environment. The proposed method (Fuzzy QE-FMEA) includes the simultaneous effects of incompatibility in view of the quality of products and processes and its impact on the natural environment. Therefore, it was assumed to include additional factors of impact on the natural environment (E), for which a new scale with triangular fuzzy numbers and ordinal numbers from 1 to 10 was developed. The impact indicator on the natural environment is integrated with the defect effect indicator (significance) indicator (Z) on the quality of the product and process, according to the so-called quality and environmental indicator (QE). Integration is carried out in a pairwise comparison matrix specially developed for this purpose. The matrix was created according to the principle of triangular fuzzy numbers. Finally, a formula is proposed to calculate for the RQE (threat priority value). Based on the RQE, the risk is determined taking into account the effects of quality incompatibility and the impact on the natural environment.

The Fuzzy QE-FMEA method was carried out for the process of five-layer stretch film for manual packaging. The test showed the effectiveness of the developed method in analysing the causes and effects of process defects, taking into account the impact on the natural environment.

The main advantages of the proposed method include:

- possibilities of determining the threats (defects) in products or processes considering simultaneously their effects and impact on the natural environment;
- reduction in uncertainty in the choice of the qualitative-environmental index (QE) by the matrix of choice for assessments for these indicators in triangular fuzzy numbers;
- improving products or processes according to the rules of continuous improvement and sustainable development;
- supporting actions of enterprises aimed at improving the quality of products and processes while simultaneously caring for the natural environment;
- predicting growth of customer satisfaction and reduction in negative impact on the natural environment.

The expected disadvantages of the proposed method include, for example, the possibility of subjectivity of expert assessments, or the need to take more improvement actions as a result of the potential increase in the RQE indicator after additionally taking into account the impact on the natural environment.

As part of future research, it is planned to test the methodology possibility of reducing the developed solely to the use of triangular fuzzy numbers. Sensitivity tests of this method will also be performed. In addition, defects in industrial products will be analysed.

The Fuzzy QE-FMEA method can be used in any enterprise to analyse any type of product or process. It means, that the Fuzzy QE-FMEA method can be used in each situation (for any problem, i.e., in the basic approach for any products or processes). However, it is necessary to consider, whether the use of this method and the solutions resulting from it always make sense. The developed method will be the most useful, for example, when the impact of products or processes on the natural environment is significant.

## Figures and Tables

**Table 1:** Characteristic of assumed priority number P—probability occurring defect of process. Own study based on [27,31,42].

Probability Occurring		The Frequency of the Defect	P	Fuzzy P	
				Tringular	Inverse
Unbelievable	the occurrence of the defect is unlikely	Less than 1/1,000,000	1	1; 1; 1	1; 1; 1
Very rarely	there are few defects	1/20,000	2	1; 2; 3	1/3; 1/2; 1
Rarely	there are relatively few defects	1/4000	3	2; 3; 4	1/4; 1/3; 1/2
On average	the defect occurs sporadically from time to time	1/1000	4	3; 4; 5	1/5; 1/4; 1/3
		1/400	5	4; 5; 6	1/6; 1/5; 1/4
		1/80	6	5; 6; 7	1/7; 1/6; 1/5
Often	the defect repeats itself cyclically	1/40	7	6; 7; 8	1/8; 1/7; 1/6
		1/20	8	7; 8; 9	1/9; 1/8; 1/7
Very often	the disadvantage is almost unavoidable	1/8	9	8; 9; 10	1/8; 1/9; 1/10
		1/2	10	10; 10; 10	1/10; 1/10; 1/10

**Table 2:** Characteristic of assumed priority number W—possibility to detect defects. Own study based on [27,31,42].

Defect Detection		W	Fuzzy W	
			Tringular	Inverse
Very low	very low probability of not detecting a defect before leaving the production process; overall automatic control; security	1–2	1; 1; 1	1; 1; 1
			1; 2; 3	1/3; 1/2; 1
Low	low probability of not detecting the defect before the end of the operation; visibility of the defect, where only some can be unidentified	3–4	2; 3; 4	1/4; 1/3; 1/2
			3; 4; 5	1/5; 1/4; 1/3
Average	average probability of not detecting the defect before the end of the operation; problematic manual control	5–6	4; 5; 6	1/6; 1/5; 1/4
			5; 6; 7	1/7; 1/6; 1/5
Medium	the average probability of not detecting a defect before the end of the operation	7–8	6; 7; 8	1/8; 1/7; 1/6
			7; 8; 9	1/9; 1/8; 1/7
High	high probability of failure to detect a defect; subjective assessment in control	9	8; 9; 10	1/8; 1/9; 1/10
Very high	very high probability of not detecting a defect; lack of product control; lack of visibility of the defect	10	10; 10; 10	1/10; 1/10; 1/10

**Table 3:** Characteristic of assumed priority number Q—the effect (significance) of the defect (so-called quality). Own study based on [27,31,42].

Effect (Significance) of the Defect (Quality)		Q	Fuzzy Q	
			Tringular	Inverse
Very small	minimal effect; lack of visibility for the client	1–2	1; 1; 1	1; 1; 1
			1; 2; 3	1/3; 1/2; 1
Small	insignificant effect; slight difficulties in functioning process; noticeable deterioration of quality	3–4	2; 3; 4	1/4; 1/3; 1/2
			3; 4; 5	1/5; 1/4; 1/3
Average	limited dissatisfaction and minor disruptions result;	5–6		
	the process does not meet the client’s expectations		4; 5; 6	1/6; 1/5; 1/4
	or is a source of nuisance;		5; 6; 7	1/7; 1/6; 1/5
	noticeable deficiencies in process quality			
Large	the result is customer dissatisfaction;	7–8	6; 7; 8	1/8; 1/7; 1/6
	process repair costs are unknown		7; 8; 9	1/9; 1/8; 1/7
Very big	the effect is very large; threatens the safety of use and violates the law	9–10	8; 9; 10	1/8; 1/9; 1/10
			10; 10; 10	1/10; 1/10; 1/10

**Table 4:** Characteristic of assumed priority number E—impact on the natural environment caused by process or product. Own study.

Impact on the Natural Environment		Q	Fuzzy Q	
			Tringular	Inverse
Negligible	the impact is practically negligible;	1	1; 1; 1	1; 1; 1
	imperceptible negative impact			
Not important	the impact is likely to be small and non-hazardous	2–3	1; 2; 3	1/3; 1/2; 1
			2; 3; 4	1/4; 1/3; 1/2
Important	the impact may be noticeable and cause limited harm or be a source of nuisance	4–6	3; 4; 5	1/5; 1/4; 1/3
			4; 5; 6	1/6; 1/5; 1/4
			5; 6; 7	1/7; 1/6; 1/5
Very important	the impact is noticeable and harmful in large quantities;	7–8	6; 7; 8	1/8; 1/7; 1/6
	it reacts to some extent with the environment and affects human health		7; 8; 9	1/9; 1/8; 1/7
Critical	the impact is destructive and causes significant harm;	9–10	8; 9; 10	1/8; 1/9; 1/10
	reacts significantly with the environment; threatens human life and health; violates the law			

**Table 5:** Values of the sum of quality and environmental impact assessments determined during the creation of the selection matrix of the quality-environmental indicator (QE).

$M_{ij}^{1}$ $=\left[a_{ij}\right]$		Impact on the Natural Environment (E_j_)									
		1	2	3	4	5	6	7	8	9	10
Quality (Q_i_)	1	2	3	4	5	6	7	8	9	10	11
	2	3	4	5	6	7	8	9	10	11	12
	3	4	5	6	7	8	9	10	11	12	13
	4	5	6	7	8	9	10	11	12	13	14
	5	6	7	8	9	10	11	12	13	14	15
	6	7	8	9	10	11	12	13	14	15	16
	7	8	9	10	11	12	13	14	15	16	17
	8	9	10	11	12	13	14	15	16	17	18
	9	10	11	12	13	14	15	16	17	18	19
	10	11	12	13	14	15	16	17	18	19	20

**Table 6:** Quotient of the sum of assessments and two cumulative assessments of quality and environmental impact.

$M_{ij}^{2}$ $=\left[b_{ij}\right]$		Impact on the Natural Environment (E_j_)									
		1	2	3	4	5	6	7	8	9	10
Quality (Q_i_)	1	1.0	1.5	2.0	2.5	3.0	3.5	4.0	4.5	5.0	5.5
	2	1.5	2.0	2.5	3.0	3.5	4.0	4.5	5.0	5.5	6.0
	3	2.0	2.5	3.0	3.5	4.0	4.5	5.0	5.5	6.0	6.5
	4	2.5	3.0	3.5	4.0	4.5	5.0	5.5	6.0	6.5	7.0
	5	3.0	3.5	4.0	4.5	5.0	5.5	6.0	6.5	7.0	7.5
	6	3.5	4.0	4.5	5.0	5.5	6.0	6.5	7.0	7.5	8.0
	7	4.0	4.5	5.0	5.5	6.0	6.5	7.0	7.5	8.0	8.5
	8	4.5	5.0	5.5	6.0	6.5	7.0	7.5	8.0	8.5	9.0
	9	5.0	5.5	6.0	6.5	7.0	7.5	8.0	8.5	9.0	9.5
	10	5.5	6.0	6.5	7.0	7.5	8.0	8.5	9.0	9.5	10.0

**Table 7:** Quotient of the sum of assessments and two cumulative assessments of quality and environmental impact expressed in triangular fuzzy numbers.

$M_{ij}^{Fuzzy}=\left[b_{ij}\right]$		Impact on the Natural Environment (E_j_)									
		1	2	3	4	5	6	7	8	9	10
Quality (Q_i_)	1	1; 1; 1	1; 2; 3	1; 2; 3	2; 3; 4	2; 3; 4	3; 4; 5	3; 4; 5	4; 5; 6	4; 5; 6	5; 6; 7
	2	1; 2; 3	1; 2; 3	2; 3; 4	2; 3; 4	3; 4; 5	3; 4; 5	4; 5; 6	4; 5; 6	5; 6; 7	5; 6; 7
	3	1; 2; 3	2; 3; 4	2; 3; 4	3; 4; 5	3; 4; 5	4; 5; 6	4; 5; 6	5; 6; 7	5; 6; 7	6; 7; 8
	4	2; 3; 4	2; 3; 4	3; 4; 5	3; 4; 5	4; 5; 6	4; 5; 6	5; 6; 7	5; 6; 7	6; 7; 8	6; 7; 8
	5	2; 3; 4	3; 4; 5	3; 4; 5	4; 5; 6	4; 5; 6	5; 6; 7	5; 6; 7	6; 7; 8	6; 7; 8	7; 8; 9
	6	3; 4; 5	3; 4; 5	4; 5; 6	4; 5; 6	5; 6; 7	5; 6; 7	6; 7; 8	6; 7; 8	7; 8; 9	7; 8; 9
	7	3; 4; 5	4; 5; 6	4; 5; 6	5; 6; 7	5; 6; 7	6; 7; 8	6; 7; 8	7; 8; 9	7; 8; 9	8; 9; 10
	8	4; 5; 6	4; 5; 6	5; 6; 7	5; 6; 7	6; 7; 8	6; 7; 8	7; 8; 9	7; 8; 9	8; 9; 10	8; 9; 10
	9	4; 5; 6	5; 6; 7	5; 6; 7	6; 7; 8	6; 7; 8	7; 8; 9	7; 8; 9	8; 9; 10	8; 9; 10	10; 10; 10
	10	5; 6; 7	5; 6; 7	6; 7; 8	6; 7; 8	7; 8; 9	7; 8; 9	8; 9; 10	8; 9; 10	10; 10; 10	10; 10; 10

**Table 8:** Matrix to select qualitative-environmental indicator (QE) in ordinal scale.

$M_{ij}^{QE}$		Impact on the Natural Environment (E_j_)									
		1	2	3	4	5	6	7	8	9	10
Quality (Q_i_)	1	1	2	2	3	3	4	4	5	5	6
	2	2	2	3	3	4	4	5	5	6	6
	3	2	3	3	4	4	5	5	6	6	7
	4	3	3	4	4	5	5	6	6	7	7
	5	3	4	4	5	5	6	6	7	7	8
	6	4	4	5	5	6	6	7	7	8	8
	7	4	5	5	6	6	7	7	8	8	9
	8	5	5	6	6	7	7	8	8	9	9
	9	5	6	6	7	7	8	8	9	9	10
	10	6	6	7	7	8	8	9	9	10	10

**Table 9:** RQE risk level classification. Own study based on [50].

Risk Level	Range of Values
Low	〈1; 51)
Medium	〈51; 101)
High	〈101; 201)
Very high	〈201; 1000

**Table 10:** Defects of process production of five-layer stretch film for manual packaging.

Analyzed Function	Potential Incompatibility	Effects of the Defect	Causes of the Defect
Stretchability of the film	Too little stretch	Poor cargo securing	Low-quality raw material
			Incorrect setting of the technological line
		High foil consumption	Low-quality raw material
			Incorrect setting of the technological line
		Film complaints	Low-quality raw material
			Incorrect setting of the technological line
Puncture resistance	Foil that pierces too easily	Unstable load	Low-quality raw material
		A large number of holes and damage in the cargo	Low-quality raw material
		High foil consumption	Too much regranulate used
		Film complaints	Low-quality raw material
		Film complaints	Incorrect setting of the technological line
Viscosity	Unstable load	Unstable load	Poor quality of the glue used
Load stabilization (retention force)	Load instability	Unstable load	Incorrect program settings on the technological line
			Poor quality of the raw material
		Film complaints	Poor quality of the raw material
Foil appearance	Discoloration	No foil transparency	Wrong production line settings
	Welded edges of the foil	Difficulty unwinding	Wrong production line settings
		Film complaints	Poor quality of the raw material
	A large number of holes	The tearing protection of the goods	Wrong production line settings
		Film complaints	Poor quality of the raw material
			Too much regranulate used

**Table 11:** Result of the Fuzzy QE-FMEA method for the production of five-layer stretch film for manual packaging.

Analysed Function	Potential Incompatibility	Effects of the Defect	Causes of the Defect	P	W	Q	E	QE	RQE
Stretchability of the film	Too little stretch	Poor cargo securing	Low-quality raw material	6	5	4	4	4	120
			Incorrect setting of the technological line	6	6	5	4	5	180
		High foil consumption	Low-quality raw material	5	6	4	4	4	120
			Incorrect setting of the technological line	5	9	5	6	6	270
		Film complaints	Low-quality raw material	6	5	4	4	4	120
			Incorrect setting of the technological line	6	6	5	6	6	216
Puncture resistance	Foil that pierces too easily	Unstable load	Low-quality raw material	6	5	4	3	4	120
		A large number of holes and damage in the cargo	Low-quality raw material	6	5	4	6	5	150
		High foil consumption	Too much regranulate used	6	7	7	9	8	336
		Film complaints	Low-quality raw material	5	8	6	5	6	240
			Incorrect setting of the technological line	6	9	5	6	6	270
Viscosity	Unstable load	Unstable load	Poor quality of the glue used	6	5	4	7	6	180
Load stabilization (retention force)	Load instability	Unstable load	Poor quality of the raw material	7	5	2	4	3	105
			Incorrect program settings on the technological line	7	7	6	7	7	343
			Poor quality of the raw material	6	7	5	6	6	252
Foil appearance		Film complaints	Poor quality of the raw material	7	4	2	4	3	84
	Discoloration Welded edges of the foil	No foil transparency	Wrong production line settings	8	4	3	8	6	192
		Difficulty unwinding	Wrong production line settings	7	5	5	5	5	175
	A large number of holes	Film complaints	Poor quality of the raw material	7	4	7	5	6	168
		The tearing protection of the goods	Wrong production line settings	7	8	6	6	6	336
		Film complaints	Poor quality of the raw material	7	8	7	9	8	448

**Table 12:** Analysis of risk for the production process of five-layer stretch film for manual packaging.

Analysed Function	Potential Incompatibility	Effects of the Defect	Causes of the Defect	RQE	Threat Level	Improving Actions
Foil appearance	Discoloration	No foil transparency	Wrong production line settings	84	High	-
Load stabilization (retention force)	Load instability	Unstable load	Incorrect program settings on the technological line	105	High	-
Stretchability of the film	Too little stretch	Poor cargo securing	Low-quality raw material	120	High	-
		High foil consumption	Low-quality raw material	120	High	-
		Film complaints	Low-quality raw material	120	High	-
Puncture resistance	Foil that pierces too easily	Unstable load	Low-quality raw material	120	High	-
		A large number of holes and damage in the cargo	Low-quality raw material	150	High	Ongoing monitoring of raw material quality, examination of each batch of foil
Foil appearance	A large number of holes	The tearing protection of the goods	Wrong production line settings	168	High	Ongoing process monitoring
	Welded edges of the foil	Film complaints	Poor quality of the raw material	175	High	Ongoing monitoring of batches of products and raw materials
Stretchability of the film	Too little stretch	Poor cargo securing	Incorrect setting of the technological line	180	High	Ongoing foil testing
Viscosity	Unstable load	Unstable load	Poor quality of the glue used	180	High	Ongoing monitoring of the production process, laboratory tests
Foil appearance	Welded edges of the foil	Difficulty unwinding	Wrong production line settings	192	High	Ongoing process monitoring
Stretchability of the film	Too little stretch	Film complaints	Incorrect setting of the technological line	216	Very high	Ongoing foil testing
Puncture resistance	Foil that pierces too easily	Film complaints	Low-quality raw material	240	Very high	Ongoing monitoring of raw material quality
Load stabilization (retention force)	Load instability	Film complaints	Poor quality of the raw material	252	Very high	Ongoing monitoring of batches of products and raw materials
Puncture resistance	Foil that pierces too easily	Film complaints	Incorrect setting of the technological line	270	Very high	Ongoing foil testing
Stretchability of the film	Too little stretch	High foil consumption	Incorrect setting of the technological line	270	Very high	Ongoing foil testing
Puncture resistance	Foil that pierces too easily	High foil consumption	Too much regranulate used	336	Very high	Ongoing monitoring of raw material quality, examination of each batch of foil
Foil appearance	A large number of holes	Film complaints	Poor quality of the raw material	336	Very high	Ongoing monitoring of batches of products and raw materials
Load stabilization (retention force)	Load instability	Film complaints	Poor quality of the raw material	343	Very high	Ongoing monitoring of batches of products and raw materials
Foil appearance	A large number of holes	Film complaints	Too much regranulate used	448	Very high	Ongoing monitoring of raw material quality, examination of each batch of foil

**Table 13:** Comparison of results from the traditional approach of the FMEA method with the proposed approach of the Fuzzy QE-FMEA method.

Analysed Function	Potential Incompatibility	Effects of the Defect	Causes of the Defect	P	W	Q	RPN	QE	RQE
Stretchability of the film	Too little stretch	Poor cargo securing	Low-quality raw material	6	5	4	120	4	120
			Incorrect setting of the technological line	6	6	5	180	5	180
		High foil consumption	Low-quality raw material	5	6	4	120	4	120
			Incorrect setting of the technological line	5	9	5	225	6	270
		Film complaints	Low-quality raw material	6	5	4	120	4	120
			Incorrect setting of the technological line	6	6	5	180	6	216
Puncture resistance	Foil that pierces too easily	Unstable load	Low-quality raw material	6	5	4	120	4	120
		A large number of holes and damage in the cargo	Low-quality raw material	6	5	4	120	5	150
		High foil consumption	Too much regranulate used	6	7	7	294	8	336
		Film complaints	Low-quality raw material	5	8	6	240	6	240
			Incorrect setting of the technological line	6	9	5	270	6	270
Viscosity	Unstable load	Unstable load	Poor quality of the glue used	6	5	4	120	6	180
Load stabilization (retention force)	Load instability	Unstable load	Incorrect program settings on the technological line	7	5	2	70	3	105
			Incorrect program settings on the technological line	7	7	6	294	7	343
			Poor quality of the raw material	6	7	5	210	6	252
Foil appearance	Discoloration Welded edges of the foil	Film complaints	Poor quality of the raw material	7	4	2	56	3	84
		No foil transparency	Wrong production line settings	8	4	3	96	6	192
		Difficulty unwinding	Wrong production line settings	7	5	5	175	5	175
	A large number of holes	Film complaints	Poor quality of the raw material	7	4	7	196	6	168
		The tearing protection of the goods	Wrong production line settings	7	8	6	336	6	336
		Film complaints	Poor quality of the raw material	7	8	7	392	8	448

## Data Availability

Not applicable.
